# Comparison of pooled semen insemination and single colony insemination as sustainable honeybee breeding strategies

**DOI:** 10.1098/rsos.231556

**Published:** 2024-01-31

**Authors:** Manuel Du, Richard Bernstein, Andreas Hoppe

**Affiliations:** Institute for Bee Research Hohen Neuendorf, Friedrich-Engels-Strasse 32, 16540 Hohen Neuendorf, Germany

**Keywords:** instrumental insemination, pooled semen, sustainable breeding, inbreeding, genetic gain

## Abstract

Instrumental insemination of honeybees allows for two opposing breeding strategies. In single colony insemination (SCI), all drones to inseminate a queen are taken from one colony. In pooled semen insemination (PSI), sperm of many genetically diverse drones is mixed and queens are fertilized from the resulting drone pool. While SCI allows for maximum pedigree control, proponents of PSI claim to reduce inbreeding and maintain genetic variance. Using stochastic simulation studies, we compared genetic progress and inbreeding rates in small honeybee populations under SCI and PSI. Four different selection criteria were covered: estimated breeding values (EBV), phenotypes, true breeding values (TBV) and random selection. Under EBV-based truncation selection, SCI yielded 9.0% to 44.4% higher genetic gain than PSI, but had vastly increased inbreeding rates. Under phenotypical or TBV selection, the gap between SCI and PSI in terms of genetic progress narrowed. Throughout, PSI yielded lower inbreeding rates than SCI, but the differences were only substantial under EBV truncation selection. As a result, PSI did not appear as a viable breeding strategy owing to its incompatibility with modern methods of genetic evaluation. Instead, SCI is to be preferred but instead of strict truncation selection, strategies to avoid inbreeding need to be installed.

## 1. Background

For many decades, instrumental insemination has been an integral part of commercial animal breeding in nearly all agricultural species [1–3]. By contrast, to this day, instrumental insemination of honeybees is still considered a niche strategy. First instrumental inseminations of honeybee queens were successfully performed in the early twentieth century [4–6]. Since then, insemination techniques have been greatly improved and turned into a practicable tool for honeybee breeding [7–9]. Nevertheless, in most parts of the world, the actual practice of instrumental insemination is still concentrated at research institutes and has only limited influence on commercial honeybee breeding [9–11]. The small impact that instrumental insemination has had on practical breeding is juxtaposed by a host of scientific studies suggesting this form of mating control to constitute a viable breeding strategy [8,11–15]. To our knowledge, presently Poland is the only country to maintain large-scale honeybee breeding programmes that rely primarily on instrumental insemination. There, each year up to 80 000 queens are fertilized in this way [16,17].

The process of instrumental insemination in honeybees differs from that in other livestock owing to the bees’ peculiar mating biology. A honeybee colony is made up of three castes: a queen, multiple thousands of workers, and (depending on the season) several hundreds of drones. Queens and workers are female, the latter sterile, while drones are the male honeybees. Shortly after hatching, the queen performs a nuptial flight and mates on the wing with several drones from other hives. The average number of drones a queen mates with is often assumed to be 12–15 [18–22], although recent research points at higher numbers [23]. For her entire life, the queen uses the semen collected during the mating flight to fertilize eggs which then develop into (diploid) female bees. Drones, by contrast, develop from unfertilized eggs and thus are haploid.

In order to instrumentally inseminate a young queen, the queen is prevented from performing her mating flight. Typically one week after hatching, the virgin queen is taken and a total of 8−12 μl of semen from several drones is injected into her vaginal orifice [8,9]. Before and during the process, the queen is narcotized with CO_2_, which stimulates oviposition [7,9,24]. However, while these cornerstones of the insemination procedure have developed into a widely accepted standard [8], the details of instrumental insemination protocols for honeybees differ in a multitude of aspects such as semen storage times or the use of diluents [9]. One aspect that is crucial in the breeding of honeybees is the choice of drones to use for the inseminations. Here, two opposing strategies have formed over the years.

One approach is to take all drones to inseminate a queen from the same colony (but generally from different colonies for different queens) [11,15,25,26]. We call this strategy *single colony insemination* (SCI). It allows us to keep accurate pedigrees and thus facilitates the use of modern techniques of genetic evaluation, such as best linear unbiased prediction (BLUP) breeding value estimation [10,19,27]. These elaborate statistic procedures in turn enable breeders to select the best stock for producing the next generation of queens and drones.

However, some breeders and scientists fear that taking drones from only one colony to inseminate a queen may narrow the genetic variance in the population and lead to high inbreeding rates. To prevent these, they propagate to pool and homogenize the semen from numerous drones of diverse origins and to inseminate many queens with the contents of the resulting semen pool [28–30]. By these *pooled semen inseminations*, they maximize the genetic diversity of female offspring of individual queens. Furthermore, they point to studies that show an enhanced overall vitality of honeybee colonies with genetically diverse worker bees [20,31,32]. However, this technique does not allow for precise paternal pedigree information and therefore has only limited compatibility with the BLUP methodology.

In the most consequent implementation of pooled semen insemination, there is only one large drone pool from which all queens are inseminated. However, in practical applications, particularly in decentrally organized breeding programmes, a more relaxed approach with several drone pools appears more realistic. We refer to the consequent and relaxed variants of *pooled semen insemination* by the abbreviations PSI and PSI*, respectively.

Although both SCI and PSI/PSI* have individually been discussed and applied for a long time, we are not aware of any theoretical or practical studies that directly compare these breeding methods. In our study, we use computer simulations to quantify the advantages and shortcomings of the insemination systems under various honeybee breeding conditions.

## 2. Methods

### 2.1. General set-up

Honeybee breeding populations were simulated with the program BeeSim [33,34]. A population of 500 breeding colonies per year was simulated over 70 years during which queens were selected for a single trait. Since most economically important traits in honeybees, like honey production or gentleness, are commonly affected by the queen and her worker group [10,21,35], we simulated the trait to express (maternal) queen effects and (direct) worker group effects. The respective genetic variances for queen and worker group effects were $\sigma_{A,Q}^{2}=1$ and $\sigma_{A,W}^{2}=2$, whereas the residual variance was chosen to be $\sigma_{E}^{2}=4$. Two different values *r*_QW_ for the genetic correlation between queen and worker effects were covered: a weak negative correlation of *r*_QW_ = −0.18 and a medium negative correlation of *r*_QW_ = −0.53. The trait was simulated to be determined by 400 unlinked biallelic loci. While the allele frequencies followed a U-shaped *β*(0.5, 0.5)-distribution, the allele effects were modelled according to a mixture of a Laplace and a normal distribution as suggested by Esfandyari *et al*. [36]. The simulated genetic architecture was thus identical to previous simulation studies on honeybee breeding that used the finite locus model [15,33,37]. Queens were distributed on 40 apiaries on which a performance test was simulated based on the colonies’ genetics and residual variances. The thus determined phenotypes served as a basis for selection. The concrete selection criteria are explained in the following subsection.

Four different selection intensities were investigated, two on the maternal and two on the paternal selection path. The selection intensity on the maternal path was determined by the number of queens that were selected to produce the next generation. This number was chosen as either *N*_dam_ = 50 (intense selection) or *N*_dam_ = 100 (moderate selection). Selection candidates for this purpose were all 2 year old queens and all selected queens produced the same number of queen offspring, resulting in fixed sister group sizes of 10 queens (intense selection) and five queens (moderate selection), respectively. On the paternal path, queens were selected as drone producers. Unlike breeding strategies that rely on isolated (natural) mating stations, instrumental insemination allows us to select the drone producing queens directly from the breeding population [15]. Selection candidates on the paternal path were all queens aged 2 or 3 years. The number of annually selected queens on the paternal path was either *N*_sire_ = 40 (intense selection) or *N*_sire_ = 80 (moderate selection).

### 2.2. Insemination strategies and selection criteria

**Figure 1.**
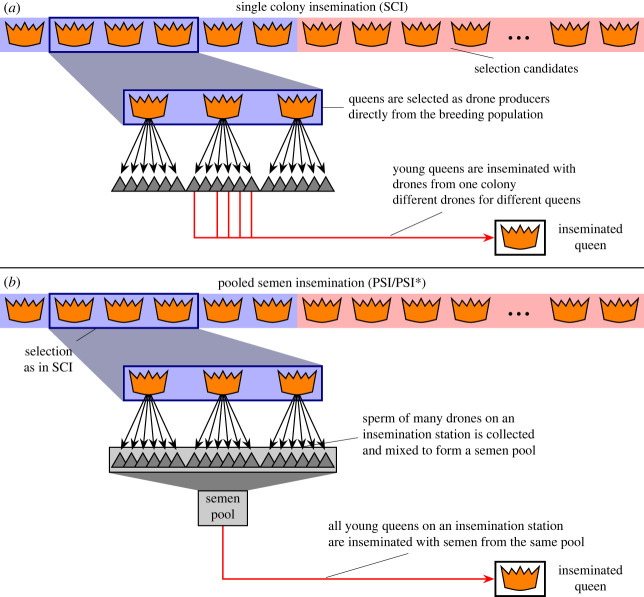
Comparison of the paternal selection paths using single colony insemination and pooled semen insemination. (*a*) is a slight variation of fig. 1 in [15], which has been published under a Creative Commons license.

Instrumental inseminations were performed according to the strategies SCI, PSI and PSI*. In each simulated population, only one insemination strategy was carried out; no mixed scenarios were investigated. In simulations with SCI (figure 1*a*), the *N*_sire_ selected drone producing queens were distributed evenly to eight separate insemination stations. Newly created queens were then sent to the insemination stations, where they were inseminated with 10 drones from a randomly chosen drone producer. However, as a measure of inbreeding avoidance, queens were not inseminated with drones from their own mother or an aunt. All virgin queens of a sister group were sent to the same randomly chosen station.

In simulations with PSI/PSI* (figure 1*b*), the selected drone producers were distributed to either one (PSI) or eight (PSI*) insemination stations. In case of PSI*, the distribution of drone producers to the insemination stations was implemented as in strategy SCI. All drone producing queens on an insemination station contributed evenly to a pool of 1000 drones. Virgin queens visiting the station were then fertilized with mixed semen from all of the 1000 drones. Thus, in strategy PSI*, depending on the intensity of paternal selection, queens were inseminated with semen of 1000 drones from five or 10 drone producing queens, while in strategy PSI the 1000 drones came from 40 or 80 different colonies.

Four different types of selection were investigated in this study: BLUP selection, phenotypic selection, selection for true breeding values (TBV selection), and random selection:

(i) in *BLUP selection*, the BLUPF90 software [38] was used to obtain maternal and direct estimated breeding values (EBV) for all queens and worker groups. According to standard practice, the selection criterion for a queen on the maternal path was defined as the sum of maternal and direct EBV of her worker group, because this value signifies the expected genetic quality of an offspring queen [19,39,40]. By contrast, when queens were selected for drone production, the genetic quality of the drones they mated with was irrelevant. Therefore, the selection criterion on this path was constituted by the sum of maternal and direct EBV of the queen herself [15,41]. The BLUP methodology requires an inverted relationship matrix for all queens and worker groups of the population. This matrix was derived from the pedigree according to the method introduced by Brascamp & Bijma [27] as implemented by Bernstein *et al.* [42]. In breeding programmes with SCI, maternal and paternal pedigree information was easily accessible. In PSI/PSI* schemes, however, it is generally not detectable from which particular drone producer a queen inherits her paternal genetics. Therefore, in PSI based breeding programmes, pedigree information was only registered on the maternal side. We expected these incomplete pedigrees to negatively impact the accuracy of breeding value estimations drastically. Therefore, we further included the following three alternative selection criteria, for which PSI/PSI* strategies do not suffer from a lack of information in comparison with SCI-based breeding schemes;
(ii) in *phenotypic selection*, queens were selected based on the observed phenotypes of their colonies. The same selection criterion thus applied for the maternal and paternal selection path;
(iii) *TBV selection* leads to the selection decisions one would make with perfect information about the bees’ genetics. As perfect information is generally not available in reality, this type of selection can only be performed *in silico*. On the maternal path, queens were selected for the sum of maternal and direct TBV of their worker groups, whereas on the paternal path, the selection criterion was formed as the sum of the queens’ own maternal and direct breeding values. The reasoning behind these different selection criteria is identical to the corresponding reasoning in BLUP selection; and
(iv) in *random selection*, queens for both selection paths were selected randomly among the respective selection candidates.

The combination of two traits, four selection intensities, three insemination strategies, and four selection criteria formed a total of 96 simulated scenarios. For each scenario, we averaged the outcomes of 100 repetitions to arrive at stable results.

### 2.3. The case of half twins

**Figure 2.**
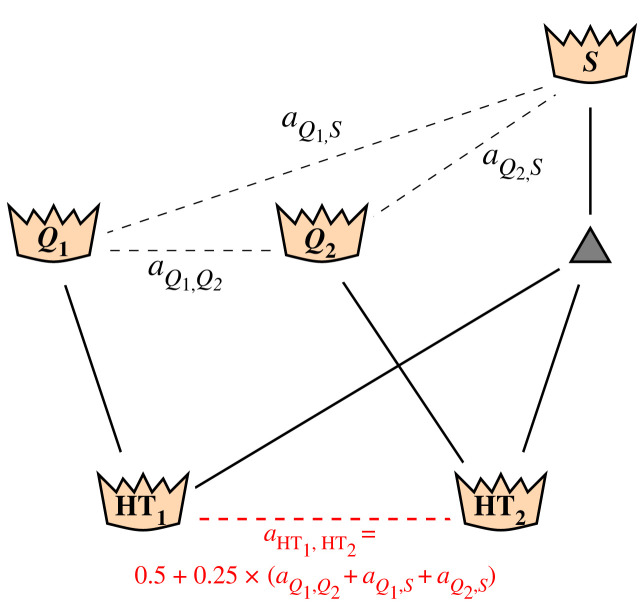
Relationship calculation between two queens with different dams who share the same father drone (half twins).

The implementation of PSI/PSI* comes with a (small) chance for a genetic oddity. If two queens are fertilized with the same semen pool, it is possible that two of their respective daughters share the same drone father [43]. Under natural mating conditions, this is impossible because drones mate only once and die immediately after ejaculation. Also in SCI as it was implemented here, this case cannot occur. The hypothetical case of two queens with different dams sharing the same father drone has previously been discussed by Ebbersten [44] who dubbed such queens *half twins*. In order to report correct inbreeding rates for all simulated scenarios, the case of two queens being half twins had to be newly implemented to BeeSim. The relationship between two half twins may be calculated according to the tabular method of Fernando & Grossmann [45], which was originally developed for X-chromosomal inheritance in mammals but can also be applied to honeybees [26,42]. Using this method to express the relationship between half twins in terms of relationships between their queen ancestors leads to the following (cf. figure 2):

let the queens HT_1_ and HT_2_ be half twins with respective dams *Q*_1_ and *Q*_2_. Further, let *S* be the producer of the common father drone of HT_1_ and HT_2_. Then the relationship $a_{{\mathrm{HT}}_{1},{\mathrm{HT}}_{2}}$ between HT_1_ and HT_2_ is calculated as

$$a_{{\mathrm{HT}}_{1},{\mathrm{HT}}_{2}}=0.5+0.25\times (a_{Q_{1},Q_{2}}+a_{Q_{1},S}+a_{Q_{2},S}),$$

where $a_{Q_{1},Q_{2}}$, $a_{Q_{1},S}$ and $a_{Q_{2},S}$ denote the (known) relationships between the respective queens.

## 3. Results

The raw results of our study are available on Dryad [34].

### 3.1. Genetic gain

**Figure 3.**
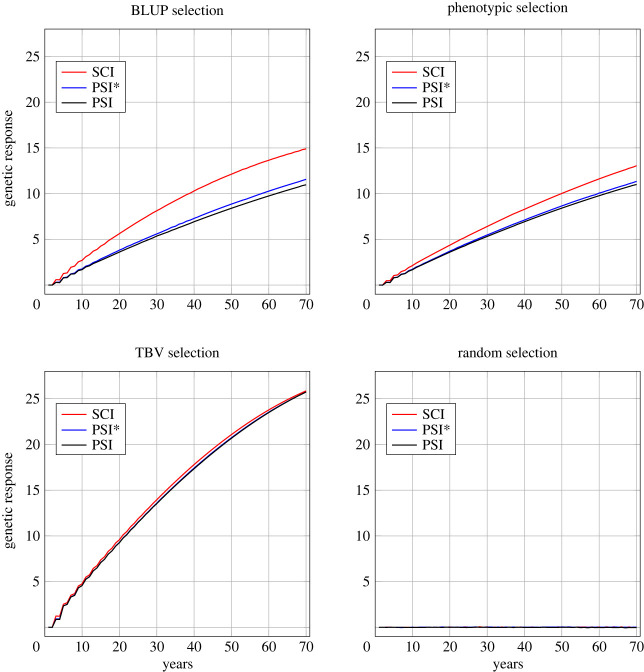
Genetic progress over 70 years for the different selection criteria and insemination strategies. Results are presented for the parameters *r*_QW_ = −0.53, *N*_dam_ = 100 and *N*_sire_ = 40.

**Figure 4.**
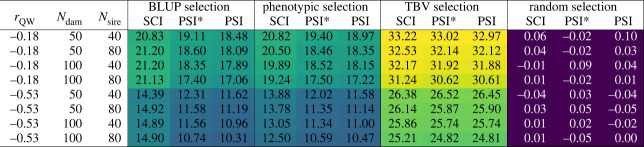
Genetic gain after 70 years in all 96 simulated scenarios.

Figure 3 displays how genetic response developed over the 70 years for the different insemination strategies and selection criteria in the situation of a trait with moderately negative correlation between queen and worker effects (*r*_QW_ = −0.53), moderate maternal selection (*N*_dam_ = 100), and strong paternal selection (*N*_sire_ = 40). Thereby, figure 3 covers 12 of the 96 simulated scenarios. These scenarios, however, are prototypical; other traits and selection intensities yielded similar graphs with only slight quantitative differences. The full set of graphs is provided in the electronic supplementary material, figure S1. Results are presented in terms of TBV in the performance criterion, i.e. the sum of queens’ maternal and worker groups’ direct breeding values [40]. A full picture of the genetic progress in all scenarios is provided by figure 4, which shows the average breeding values in the performance criterion which were achieved after 70 years of breeding.

Throughout, TBV selection achieved the highest rates of genetic improvement with between 24.81 and 33.22 units after 70 years (figure 4). By contrast, random selection did not lead to any genetic progress. With BLUP selection or phenotypic selection, after 70 years about half of the genetic gain with TBV selection could be reached (between 41.6% and 67.6%). In non-random selection strategies, the genetic progress was on average 46.2% higher for the trait with weak negative correlation between effects (*r*_QW_ = −0.18) than for the trait with moderate negative correlation (*r*_QW_ = −0.53). Mostly, more intense selection was concomitant with higher genetic gain. An exception to this rule could be observed for BLUP selection with SCI, where the strategies with moderate selection on either the maternal side (i.e. *N*_dam_ = 100) or on the paternal side (i.e. *N*_sire_ = 80) proved superior in the time frame of 70 years.

BLUP selection was always superior to phenotypic selection in terms of genetic progress after 70 years when SCI was performed (by up to 19.1%). However, the advantage was only marginal in the most intense maternal selection regiments (*N*_dam_ = 50) and *r*_QW_ = −0.18 owing to a severe flattening of the revenue curve under BLUP selection from year 40 onwards (cf. electronic supplementary material, figure S1). For insemination strategies PSI and PSI*, BLUP selection could generally not generate significantly higher genetic progress than phenotypic selection. Here, for the trait with *r*_QW_ = −0.18, phenotypic selection even yielded slight but consistent higher genetic gain.

For all non-random selection criteria, SCI yielded higher genetic gain than both PSI and PSI*, the difference between PSI and PSI* being marginal. The gap between SCI and PSI/PSI* was small for TBV selection (less than 1% on average), wider for phenotypic selection (7.3–23.7%) and most pronounced for BLUP selection (9.0–44.4%).

### 3.2. Inbreeding

**Figure 5.**
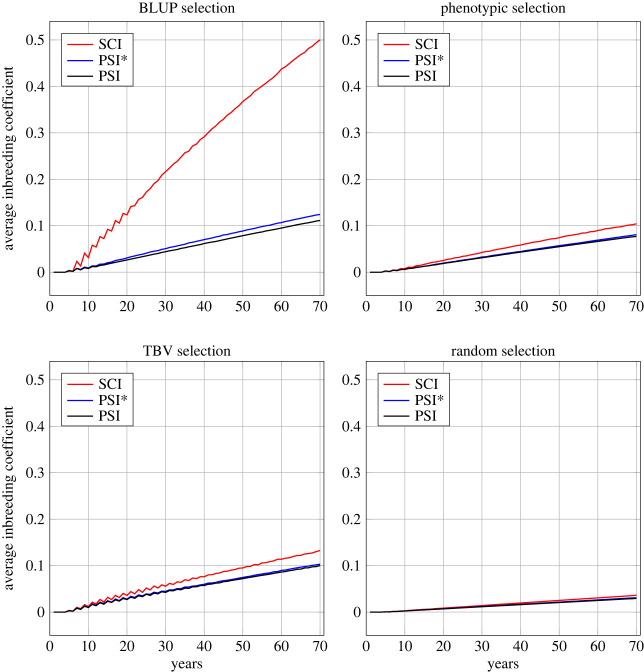
Progression of average inbreeding coefficients over 70 years for the different selection criteria and insemination strategies. Results are presented for the parameters *r*_QW_ = −0.53, *N*_dam_ = 100 and *N*_sire_ = 40.

**Figure 6.**
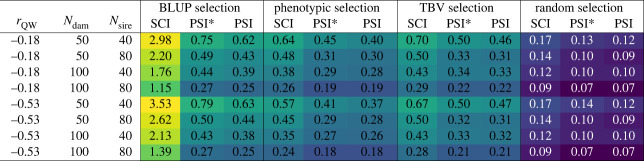
Inbreeding rates (increase of average inbreeding per generation) all 96 simulated scenarios. Results are given in percentages.

Figure 5 shows how the average inbreeding coefficients progressed over the 70 years for the different insemination strategies and selection criteria in the same situation as figure 3 had shown for the genetic gain (i.e. *r*_QW_ = −0.53, *N*_dam_ = 100, *N*_sire_ = 40). Again, these scenarios are prototypical and the inbreeding development for other traits and selection intensities was similar (electronic supplementary material, figure S2). Figure 6 shows the inbreeding rates for all scenarios, i.e. the average increase of inbreeding coefficients per generation.

Random selection yielded the lowest inbreeding rates (on average 0.11% per generation), followed by phenotypic selection (0.33%), TBV selection (0.38%) and BLUP selection (1.05%) (figure 6). In BLUP selection, we observed vast differences between insemination strategies SCI and PSI/PSI*. While SCI led to inbreeding rates between 1.15% and 3.53% per generation, the inbreeding rates for strategies PSI and PSI* with BLUP selection only ranged from 0.25% to 0.79%. Also in the other selection strategies, SCI generated higher inbreeding rates than PSI/PSI*, but the differences were much smaller (only 0.02–0.24%). The differences between PSI and PSI* were negligible, with minimally higher inbreeding rates for PSI*. With the exception of the combination of SCI and BLUP selection, all inbreeding rates were below 1% per generation.

For all selection criteria and insemination strategies, intensified selection yielded higher inbreeding rates. Hereby, halving the number of dams generally had a greater effect than halving the number of sires. The influence of the genetic parameters on inbreeding rates differed for the different selection criteria. The weaker negative correlation between effects (*r*_QW_ = −0.18) led to lower inbreeding rates in BLUP selection, but higher inbreeding rates in phenotypic selection and TBV selection. For random selection, the trait parameters did not show any influence on inbreeding.

## 4. Discussion

### 4.1. Genetic progress and inbreeding

Our general findings that phenotypic and BLUP selection yielded about half of the genetic progress as TBV selection, while random selection did not generate any progress are in good agreement with results of Quinton & Smith [46], who performed a simulation study on mammals with the same modes of selection. They also found that BLUP selection yielded the highest and random selection the lowest inbreeding rates. However, unlike in our study, where TBV selection clearly led to higher inbreeding than phenotypic selection, their results showed no significant differences between these selection criteria in terms of inbreeding. The observation that a stronger negative correlation *r*_QW_ between maternal and direct effects leads to lower genetic gain is caused by a resulting lower heritability. It is a standard finding of quantitative genetics and has shown consistently in numerous studies on honeybees [26,33,47] and other agricultural species [48–50]. In a simulation study on honeybee selection with mating control via isolated mating stations, [51] we previously found that BLUP selection in comparison to phenotypic selection generated higher genetic progress at the cost of increased inbreeding rates. Furthermore, the gaps between both selection strategies in terms of genetic progress and inbreeding were wider for lower heritabilities (i.e. stronger negative *r*_QW_). This corresponds to the observations we made with SCI.

The comparison of results for SCI and PSI/PSI* confirmed our initial expectation that PSI and BLUP are not compatible. In many cases, the incompatibility even reached an extent that BLUP selection proved inferior to phenotypic selection. In a study on uncontrolled mating in honeybees, Plate *et al.* [52] showed that the absence of paternal pedigree information under natural mating leads to severe biases in the estimation of maternal and direct breeding values. It is likely that these biases prevailed under PSI/PSI*.

Our study corroborated the assumptions of several authors that PSI leads to larger effective population sizes and thus reduced inbreeding rates [29,30,53]. However, the effect was only large under BLUP selection, not under the other selection criteria. Some authors claim PSI/PSI* to be superior in maintaining genetic diversity and avoiding inbreeding simply because of the great diversity of drones that are used to inseminate a queen [54]. This assessment is too superficial. While it is true that the direct offspring of a queen is genetically diverse, there are no increased genetic differences between offspring of different queens. The potential to maintain genetic variance and avoid inbreeding over multiple generations is therefore limited.

For all simulated selection intensities, BLUP selection in combination with SCI yielded inbreeding rates above 1% per generation. According to the definition of the Food and Agriculture Organisation of the United Nations (FAO), these selection strategies are thus to be considered unsustainable [15,37,55]. By contrast, all selection schemes that relied on other forms of selection or on PSI/PSI* were sustainable.

### 4.2. Implications for sustainable honeybee breeding

Our results clearly indicate that under the assumed parameters, BLUP truncation selection with SCI is no sustainable breeding alternative. This is in line with our earlier findings in [15], where we showed that under moderate maternal selection (*N*_dam_ = 100), the required number of sires for sustainable BLUP truncation selection is at least *N*_sire_ = 160, whereas this study only covered the smaller options *N*_sire_ = 40 and *N*_sire_ = 80. In contrast to isolated (natural) mating stations, instrumental insemination allows for great flexibility so that in many cases it will easily be possible to increase the number of selected drone producing queens in order to achieve acceptable inbreeding rates.

However, even in cases where the possible number *N*_sire_ of selected drone producers is restricted, our results do not indicate that one should dismiss BLUP breeding value estimation as a means of genetic evaluation in favour of phenotypic selection. Instead, one should deviate from a strict truncation selection strategy and follow measures of inbreeding avoidance such as within-family selection [26,56]. In fact, we are not aware of any real honeybee breeding scheme that implements strict truncation selection on a closed population. Instead, breeders follow diverse breeding goals [10], regularly introduce new stock into the population [21,57] or perform within-family selection [26].

The honeybee breeding database www.beebreed.eu allows us to precalculate inbreeding rates of offspring resulting from different mating options. While this tool can effectively be used to avoid inbreeding in the short term [58], an effective long-term inbreeding reduction under BLUP breeding value estimation probably requires more sophisticated strategies such as optimal contribution selection [59] or the assumption of genetic parameters with overestimated heritabilities in the BLUP procedure [51,60].

In contrast to SCI, PSI/PSI* currently only allow for primitive methods of genetic evaluation, such as phenotypic selection. However, when these are applied, the differences to SCI schemes in terms of inbreeding are small. Thus, under current conditions, PSI/PSI* appears as an inferior strategy for honeybee breeding. Nevertheless, the results for TBV selection show that if only one had sufficient information on the breeding colonies’ genetics, PSI/PSI* could be turned into a viable alternative insemination strategy. However, to date, this is hampered by the loss of paternal pedigree information.

With the advent of genomic selection strategies, also for honeybees [61–63], the prospect of PSI/PSI* strategies might become slightly brighter, because with genomic information, the importance of pedigrees is diminished. Realistically, however, even in populations where genomic selection is applied, only a fraction of colonies (and only the queens) will be genotyped [64], so that reliable pedigree information remains crucial for successful BLUP evaluations. It might be possible to use genomic information to reconstruct pedigrees also in case of PSI/PSI* by comparing genotypes between the individual drone producers and their paternal offspring, similar to the strategies presented in [65]. However, such approaches are not yet fully worked out and it still requires considerable theoretical work to make them actually feasible.

### 4.3. Breeding stock versus commercial stock

Proponents of PSI might object that we did not account for the increased fitness of colonies that result from the increased genetic diversity among the workers [20,31,32]. Indeed, this increased fitness probably also influences the performances in economically important traits like honey yield. However, these diversity effects are not heritable and queen offspring of a pooled-semen inseminated queen will have no genetic benefit from the enhanced diversity of their dam’s worker group [15,58]. Thus, even when accounting for these fitness benefits, no higher rates of genetic progress are to be expected.

Nevertheless, the fitness advantages that result from PSI can be exploited in honeybee production schemes. This exploitation requires a differentiation between the *breeding value* and the *commercial value* of a colony. The commercial value of a colony is directly defined by its performance, i.e. the amount of honey produced or the resistance against *Varroa destructor* or other pests, and can very well be influenced by the genetic diversity among the worker bees. By contrast, the breeding value is defined by the ability to transmit genetic qualities to the next generation and is independent of properties of the (non-reproducing) workers. A prosperous honeybee production strategy may thus consist of breeding with SCI and finally sell commercial stock consisting of a last generation of queens that were inseminated with pooled semen. This procedure is similar to the standard protocols in pig breeding, where the breeding stock is outcrossed in the last generation to make use of heterosis effects in the commercial stock [66]. It should be noted, however, that this strategy assumes that queens bred to perform well under SCI will also perform well when being inseminated with a semen pool. In other words, the traits *performance under SCI* and *performance under PSI* need to be highly correlated. In cross-breeding schemes for pigs, this assumption is not always met but strategies to circumvent the problem have been developed [67]; for honeybees, we are not aware of any studies in this direction. Presently, the differentiation between breeding stock and commercial stock in honeybees is not sufficiently internalized by most breeders.

## Data Availability

The program BeeSim and the simulation data are available from the Dryad Digital Repository: http://doi.org/10.5061/dryad.stqjq2c8t [34]. Two supplementary figures are provided in the electronic supplementary material [68].
